# 1-Methyl-4-(4-nitro­benzo­yl)pyridinium perchlorate

**DOI:** 10.1107/S1600536811034945

**Published:** 2011-08-31

**Authors:** Tobias Gruber, Frank Eissmann, Edwin Weber, Gerrit Schüürmann

**Affiliations:** a UFZ Department of Ecological Chemistry, Helmholtz Centre for Environmental Research, Permoserstrasse 15, D-04318 Leipzig, Germany; b Institut für Organische Chemie, TU Bergakademie Freiberg, Leipziger Strasse 29, D-09596 Freiberg/Sachsen, Germany

## Abstract

In the main mol­ecule of the title compound, C_13_H_11_N_2_O_3_^+^·ClO_4_^−^, the two aromatic rings are twisted by 56.19 (3)° relative to each other and the nitro group is not coplanar with the benzene ring [36.43 (4)°]. The crystal packing is dominated by infinite aromatic stacks in the *a-*axis direction. These are formed by the benzene units of the mol­ecule featuring an alternating arrangement, which explains the two different distances of 3.3860 (4) and 3.4907 (4) Å for the aromatic units (these are the perpendicular distances of the centroid of one aromatic ring on the mean plane of the other other aromatic ring). Adjacent stacks are connected by π–π stacking between two pyridinium units [3.5949 (4) Å] and weak C—H⋯O inter­actions. The perchlorate anions are accomodated in the lattice voids connected to the cation *via* weak C—H⋯O contacts between the O atoms of the anion and various aromatic as well as methyl H atoms.

## Related literature

For an alternative synthesis and the electrochemical and host/guest characteristics of the title compound, see: Fischer (1973 ▶); Leventis *et al.* (2004*a* ▶,*b* ▶); Rawashdeh *et al.* (2008 ▶). For related pyridinium ions, see: Kolev *et al.* (2001 ▶, 2005 ▶, 2006 ▶). For complexes of 4-benzoyl­pyridine with transistion metals, see: Araki *et al.* (2005 ▶); Mautner & Gohera (1998 ▶); Gohera & Mak (1998 ▶); Escuer *et al.* (2000 ▶); Gohera & Mautner (1999 ▶); Drew *et al.* (1985 ▶); Gotsis & White (1987 ▶). Respective co-crystals and derivatives are discussed in Sugiyama *et al.* (2002*a* ▶,*b* ▶) and Syed *et al.* (1984 ▶).
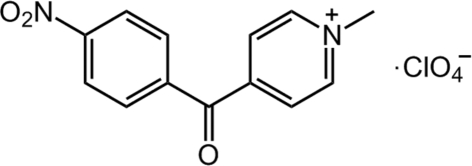


## Experimental

### 

#### Crystal data

C_13_H_11_N_2_O_3_^+^·ClO_4_^−^
*M* *_r_* = 342.69
Triclinic, 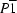

*a* = 7.9240 (3) Å
*b* = 7.9800 (3) Å
*c* = 12.6350 (6) Å
α = 105.980 (2)°
β = 104.119 (1)°
γ = 99.138 (1)°
*V* = 722.67 (5) Å^3^
*Z* = 2
Mo *K*α radiation
μ = 0.31 mm^−1^
*T* = 153 K
0.45 × 0.39 × 0.15 mm

#### Data collection

Bruker Kappa APEXII CCD diffractometer
Absorption correction: multi-scan (*SADABS*; Bruker, 2007 ▶) *T*_min_ = 0.875, *T*_max_ = 0.919
20599 measured reflections
5199 independent reflections
4759 reflections with *I* > 2σ(*I*)
*R*_int_ = 0.019

#### Refinement

*R*[*F* ^2^ > 2σ(*F* ^2^)] = 0.031
*wR*(*F* ^2^) = 0.092
*S* = 1.06
5199 reflections
209 parameters
H-atom parameters constrained
Δρ_max_ = 0.65 e Å^−3^
Δρ_min_ = −0.46 e Å^−3^

### 

Data collection: *APEX2* (Bruker, 2007 ▶); cell refinement: *SAINT* (Bruker, 2007 ▶); data reduction: *SAINT*; program(s) used to solve structure: *SHELXS97* (Sheldrick, 2008 ▶); program(s) used to refine structure: *SHELXL97* (Sheldrick, 2008 ▶); molecular graphics: *SHELXTL* (Sheldrick, 2008 ▶) and *ORTEP-3* (Farrugia, 1997 ▶); software used to prepare material for publication: *SHELXTL* and *PLATON* (Spek, 2009 ▶).

## Supplementary Material

Crystal structure: contains datablock(s) global, I. DOI: 10.1107/S1600536811034945/im2313sup1.cif

Structure factors: contains datablock(s) I. DOI: 10.1107/S1600536811034945/im2313Isup2.hkl

Supplementary material file. DOI: 10.1107/S1600536811034945/im2313Isup3.cml

Additional supplementary materials:  crystallographic information; 3D view; checkCIF report

## Figures and Tables

**Table 1:** Hydrogen-bond geometry (Å, °)

*D*—H⋯*A*	*D*—H	H⋯*A*	*D*⋯*A*	*D*—H⋯*A*
C2—H2⋯O7^i^	0.95	2.60	3.478 (1)	153
C3—H3⋯O7^ii^	0.95	2.42	3.277 (1)	150
C5—H5⋯O4	0.95	2.51	3.394 (1)	154
C9—H9⋯O7^iii^	0.95	2.42	3.132 (1)	132
C10—H10⋯O3^iv^	0.95	2.52	3.392 (1)	153
C11—H11⋯O1^v^	0.95	2.43	3.200 (1)	138
C12—H12⋯O5^vi^	0.95	2.39	3.134 (1)	135
C13—H13*A*⋯O3^iv^	0.98	2.63	3.373 (1)	133
C13—H13*B*⋯O6^vii^	0.98	2.59	3.429 (1)	144
C13—H13*C*⋯O2^viii^	0.98	2.63	3.446 (1)	141
C13—H13*C*⋯O6	0.98	2.60	3.402 (1)	139

Symmetry codes: (i) 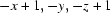; (ii) 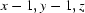; (iii) 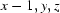; (iv) 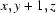; (v) 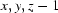; (vi) 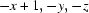; (vii) 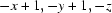; (viii) 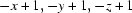.

**Fig. 1.**
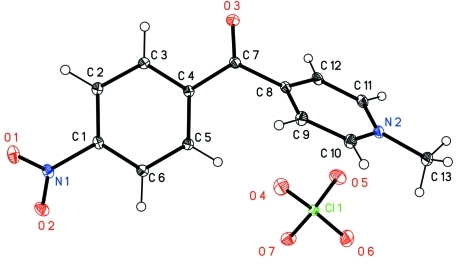
Molecular structure of the title compound with 50% probability displacement ellipsoids.

**Fig. 2.**
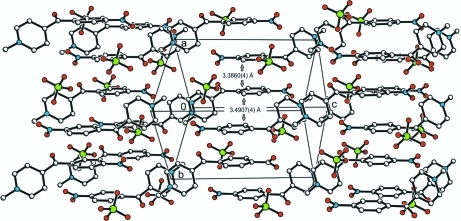
Packing diagram of (I). Hydrogen atoms have been omitted for clarity.

**Fig. 3.**
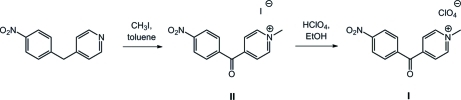
Synthesis scheme.

## supplementary crystallographic information

### Comment

The development of nonlinear optical materials has attracted much attention in the last years. A typical representative consists of conjugated π-systems in which nonlinearities can be achieved by introduction of donor and acceptor substituents. A compound family with such properties relates to appropriately derivatized 4-benzoylpyridine and its respective pyridinium ions (Kolev *et al.*, 2006), with the title compound (I) as an example. Its electrochemical (Leventis *et al.*, 2004*a*,*b*) and host/guest (Rawashdeh *et al.*, 2008) characteristics have already been reported earlier. However, considering research on the behaviour in the crystal state, only 4-benzoylpyridine as a mother compound was described so far in complexes with transition metals (Araki *et al.*, 2005; Mautner & Gohera, 1998; Gohera & Mak, 1998; Escuer *et al.*, 2000; Gohera & Mautner, 1999; Drew *et al.*, 1985; Gotsis & White, 1987) as well as in co-crystals with various benzoic acids (Sugiyama *et al.*, 2002*a*,*b*) and being derivatized with chlorine in the *para* position of the benzene ring (Syed *et al.*, 1984). Structural studies on the respective benzoyl-pyridinium species are rather rare both featuring a squaric acid group at the nitrogen atom (Kolev *et al.*, 2001; Kolev *et al.*, 2005). As an extension to the literature, we present in this paper the synthesis and structure characteristics of *N*-methyl-4-(4-nitrobenzoyl)pyridinium perchlorate (I).

Compound (I) crystallizes from a mixture of ethanol and perchloric acid (20:3 *v*/*v*) as colourless crystals in the triclinic space group *P*-1 with one cation and one anion in the asymmetric unit (Fig. 1). No solvent is included in the crystal structure. In the perchlorate anion, the Cl—O bond distances [1.4337 (8)–1.4401 (8) Å] and O—Cl—O bond angles [108.89 (6)–110.12 (6)°] confirm a tetrahedral configuration. Considering the cation, the aromatic and the pyridinium ring are more or less planar with atoms C3 and C8 deviating as much as 0.0115 (6) and 0.0183 (6) Å from their respective meanplanes. Furthermore, the nitro group is not completely coplanar to the corresponding benzene ring [36.43 (4)°]. As anticipated, the central carbonyl part of the structure shows a high degree of planarity, though the overall cation adopts a twisted conformation to minimalize the repulsion between its two rings: torsion angles C5—C4—C7—C8 and C4—C7—C8—C9 are -23.70 (11) and -42.06 (11)°, respectively, and we observed a dihedral angle for the two rings of 56.19 (3)°.

The title compound lacks of donors for strong hydrogen bonds, thus the crystal packing is dominated by aromatic stacks in direction of the crystallographic *a* axis. These are formed by the slightly displaced and tilted benzene units of the molecule featuring an alternating arrangement, which explains the two different distances of 3.3860 (4) and 3.4907 (4) Å for the aromatic units (Fig. 2). Similar to the dimeric structure of the 4-benzoylpyridine in its monoprotonated form (Mautner & Gohera, 1998), adjacent stacks are connected by π-π-stacking between two pyridinium units [d = 3.5949 (4) Å] and weak C—H···O interactions [*d*(H···O) = 2.43–2.63 Å] involving two of the aromatic H atoms (H10, H11) and two methyl H atoms (H13A, H13C) on the one hand and the carbonyl oxygen (O3) as well as the two nitro O atoms (O1, O2) on the other hand. The perchlorate anions are accommodated in the lattice voids connected to the cation *via* weak C—H···O contacts between the O atoms of the anion and various aromatic as well as methyl H atoms.

In conclusion, the title compound, similar to the related compounds, shows a twisted conformation in the crystalline state [56.19 (3)°], in order to avoid sterical clash. It is interesting to note that another substituent at the benzene unit [*p*-chlorobenzoylpyridine (Syed *et al.*, 1984)] produces a more similar dihedral angle (52.2°) than observed for the squaric acid derivative of benzoyl pyridinium (82.6°) (Kolev *et al.*, 2005). Compared to them, the torsion angles of the title compound reveal a much higher twist of the carbonyl group and the two adjacent rings. Further investigation on the influence of different substituents at the benzene and pyridine entities will deliver more information about this interesting class of compounds.

### Experimental

Following a procedure for the synthesis of *N*-methyl-4-(4-nitrobenzyl)pyridinium iodide described by Fischer (1973), we obtained the respective benzoyl species in a two-step synthesis.

To a stirred solution of 2.14 g (10 mmol) 4-(4-nitrobenzyl)pyridine in 20 ml toluene, 2.50 g (17.6 mmol) methyl iodide were added. While heating under reflux for 30 min, the colour of the solution changed from yellow to purple, and a solid precipitated, which was collected and recrystallized from acetone/methanol (1:1 *v*/*v*). After several days, *N*-Methyl-4-(4-nitrobenzoyl)pyridinium iodide could be harvested as deep red crystals (1.65 g, 45%). *M*.p. 484–485 K. A solution of 1.0 g (2.7 mmol) of *N*-Methyl-4-(4-nitrobenzoyl)pyridinium iodide in 100 ml ethanol was reacted with 15 ml perchloric acid (70%) (Caution!) to yield colourless crystals of the title compound after four weeks (230 mg, 25%). *M*.p. 464–465 K.

### Refinement

H atoms were positioned geometrically and allowed to ride on their respective parent atoms, with C—H = 0.98 Å and *U*_iso_(H) = 1.5*U*_eq_(C) for methyl, and C—H = 0.95 Å and *U*_iso_(H) = 1.2*U*_eq_(C) for aryl H atoms.

### Crystal data

**Table:**

C_13_H_11_N_2_O_3_^+^·ClO_4_^−^	*Z* = 2
*M**_r_* = 342.69	*F*(000) = 352
Triclinic, *P*1	*D*_x_ = 1.575 Mg m^−^^3^
Hall symbol: -P 1	Mo *K*α radiation, λ = 0.71073 Å
*a* = 7.9240 (3) Å	Cell parameters from 6858 reflections
*b* = 7.9800 (3) Å	θ = 2.7–44.1°
*c* = 12.6350 (6) Å	µ = 0.31 mm^−^^1^
α = 105.980 (2)°	*T* = 153 K
β = 104.119 (1)°	Piece, colourless
γ = 99.138 (1)°	0.45 × 0.39 × 0.15 mm
*V* = 722.67 (5) Å^3^	

### Data collection

**Table:**

Bruker Kappa APEXII CCD diffractometer	5199 independent reflections
Radiation source: fine-focus sealed tube	4759 reflections with *I* > 2σ(*I*)
graphite	*R*_int_ = 0.019
φ and ω scans	θ_max_ = 32.5°, θ_min_ = 2.8°
Absorption correction: multi-scan (*SADABS*; Bruker, 2007)	*h* = −11→11
*T*_min_ = 0.875, *T*_max_ = 0.919	*k* = −12→12
20599 measured reflections	*l* = −19→19

### Refinement

**Table:**

Refinement on *F*^2^	Primary atom site location: structure-invariant direct methods
Least-squares matrix: full	Secondary atom site location: difference Fourier map
*R*[*F*^2^ > 2σ(*F*^2^)] = 0.031	Hydrogen site location: inferred from neighbouring sites
*wR*(*F*^2^) = 0.092	H-atom parameters constrained
*S* = 1.06	*w* = 1/[σ^2^(*F*_o_^2^) + (0.0551*P*)^2^ + 0.1728*P*] where *P* = (*F*_o_^2^ + 2*F*_c_^2^)/3
5199 reflections	(Δ/σ)_max_ = 0.001
209 parameters	Δρ_max_ = 0.65 e Å^−^^3^
0 restraints	Δρ_min_ = −0.46 e Å^−^^3^

### Special details

**Table:**

Experimental. *N*-Methyl-4-(4-nitrobenzoyl)pyridinium iodide (II). To a stirred solution of 2.14?g (10?mmol) 4-(4-nitrobenzyl)pyridine in 20?ml toluene, 2.50 g (17.6 mmol) methyl iodide were added. While heating under reflux for 30 min, the colour of the solution changed from yellow to purple, and a solid precipitated, which was collected and recrystallized from acetone/methanol (1:1 v/v). After several days, II could be harvested as deep red crystals (1.65 g, 45%). M.p. 484–485 K. 1H-NMR (DMSO-d6) δ 4.50 (s, 3 H, CH3), 8.10 (d, 2 H, ArH-9, ArH-12), 8.45 (m, 4 H, ArH-2, ArH-3, ArH-5, ArH-6), 9.28 (d, 2 H, ArH-10, ArH-11); 13C-NMR (DMSO-d6) δ 48.44 (CH3), 123.92, 126.84 (2-, 6-, 9-, 12-ArC), 131.60 (3-, 5-ArC), 139.13 (4-ArC), 146.66 (8-ArC), 149.73, 150.44 (1-, 10-, 11-ArC), 191.03 (C=O).*N*-Methyl-4-(4-nitrobenzoyl)pyridinium perchlorate (I). A solution of 1.0 g (2.7 mmol) of II in 100 ml ethanol was reacted with 15 ml perchloric acid (70%) (Caution!) to yield colourless crystals of (I) after four weeks (230 mg, 25%). M.p. 464–465 K. 1H-NMR (DMSO-d6) δ 4.46 (s, 3 H, CH3), 8.06 (d, 2 H, ArH-9, ArH-12), 8.28 (m, 4 H, ArH-2, ArH-3, ArH-5, ArH-6), 9.21 (d, 2 H, ArH-10, ArH-11); 13C-NMR (DMSO-d6) δ 48.33 (CH3), 123.98, 126.91 (2-, 6-, 9-, 12-ArC), 131.57 (3-, 5-ArC), 139.22 (4-ArC), 146.70 (8-ArC), 149.84, 150.54 (1-, 10-, 11-ArC), 191.08 (C=O).
Geometry. All e.s.d.'s (except the e.s.d. in the dihedral angle between two l.s. planes) are estimated using the full covariance matrix. The cell e.s.d.'s are taken into account individually in the estimation of e.s.d.'s in distances, angles and torsion angles; correlations between e.s.d.'s in cell parameters are only used when they are defined by crystal symmetry. An approximate (isotropic) treatment of cell e.s.d.'s is used for estimating e.s.d.'s involving l.s. planes.
Refinement. Refinement of *F*^2^ against ALL reflections. The weighted *R*-factor *wR* and goodness of fit *S* are based on *F*^2^, conventional *R*-factors *R* are based on *F*, with *F* set to zero for negative *F*^2^. The threshold expression of *F*^2^ > σ(*F*^2^) is used only for calculating *R*-factors(gt) *etc*. and is not relevant to the choice of reflections for refinement. *R*-factors based on *F*^2^ are statistically about twice as large as those based on *F*, and *R*- factors based on ALL data will be even larger.

### Fractional atomic coordinates and isotropic or equivalent isotropic displacement parameters (Å^2^)

**Table:**

	*x*	*y*	*z*	*U*_iso_*/*U*_eq_	
O1	0.21076 (10)	0.03649 (11)	0.75601 (6)	0.02567 (14)	
O2	0.48688 (9)	0.15551 (10)	0.77549 (6)	0.02398 (14)	
O3	0.09175 (9)	−0.25751 (9)	0.16586 (6)	0.02171 (13)	
N1	0.33202 (10)	0.07804 (10)	0.71642 (6)	0.01796 (13)	
N2	0.23169 (10)	0.29598 (10)	0.06813 (6)	0.01774 (13)	
C1	0.28772 (11)	0.03276 (11)	0.59073 (7)	0.01597 (13)	
C2	0.16321 (11)	−0.12663 (11)	0.52203 (7)	0.01882 (15)	
H2	0.1083	−0.2049	0.5553	0.023*	
C3	0.12131 (11)	−0.16827 (11)	0.40303 (7)	0.01830 (14)	
H3	0.0389	−0.2780	0.3535	0.022*	
C4	0.20033 (11)	−0.04878 (10)	0.35605 (7)	0.01519 (13)	
C5	0.32546 (11)	0.11108 (11)	0.42782 (7)	0.01684 (14)	
H5	0.3786	0.1914	0.3952	0.020*	
C6	0.37179 (11)	0.15206 (11)	0.54715 (7)	0.01746 (14)	
H6	0.4585	0.2587	0.5973	0.021*	
C7	0.15140 (10)	−0.10129 (11)	0.22792 (7)	0.01583 (13)	
C8	0.17711 (10)	0.04177 (11)	0.17269 (7)	0.01528 (13)	
C9	0.13320 (12)	0.20531 (11)	0.21083 (7)	0.01856 (15)	
H9	0.0845	0.2303	0.2735	0.022*	
C10	0.16116 (12)	0.33074 (12)	0.15649 (8)	0.01980 (15)	
H10	0.1305	0.4423	0.1815	0.024*	
C11	0.27066 (12)	0.13678 (12)	0.02789 (7)	0.01927 (15)	
H11	0.3181	0.1145	−0.0354	0.023*	
C12	0.24204 (12)	0.00559 (12)	0.07807 (7)	0.01801 (14)	
H12	0.2664	−0.1079	0.0483	0.022*	
C13	0.27377 (13)	0.43875 (13)	0.01795 (8)	0.02376 (17)	
H13A	0.1777	0.5026	0.0122	0.036*	
H13B	0.2839	0.3847	−0.0592	0.036*	
H13C	0.3875	0.5235	0.0676	0.036*	
Cl1	0.70498 (3)	0.38019 (2)	0.243705 (16)	0.01746 (6)	
O4	0.58098 (11)	0.28582 (13)	0.28579 (8)	0.03593 (19)	
O5	0.69466 (14)	0.27089 (12)	0.12976 (7)	0.0388 (2)	
O6	0.66108 (14)	0.54567 (11)	0.23798 (8)	0.0376 (2)	
O7	0.88363 (10)	0.41729 (10)	0.32064 (7)	0.02936 (16)	

### Atomic displacement parameters (Å^2^)

**Table:**

	*U*^11^	*U*^22^	*U*^33^	*U*^12^	*U*^13^	*U*^23^
O1	0.0294 (3)	0.0341 (4)	0.0188 (3)	0.0093 (3)	0.0123 (3)	0.0115 (3)
O2	0.0234 (3)	0.0258 (3)	0.0179 (3)	0.0067 (2)	−0.0007 (2)	0.0054 (2)
O3	0.0258 (3)	0.0173 (3)	0.0178 (3)	0.0018 (2)	0.0047 (2)	0.0028 (2)
N1	0.0216 (3)	0.0198 (3)	0.0142 (3)	0.0086 (2)	0.0048 (2)	0.0068 (2)
N2	0.0178 (3)	0.0209 (3)	0.0143 (3)	0.0026 (2)	0.0037 (2)	0.0078 (2)
C1	0.0170 (3)	0.0192 (3)	0.0127 (3)	0.0059 (3)	0.0044 (2)	0.0059 (3)
C2	0.0205 (3)	0.0196 (3)	0.0166 (3)	0.0020 (3)	0.0055 (3)	0.0081 (3)
C3	0.0196 (3)	0.0173 (3)	0.0159 (3)	0.0003 (3)	0.0041 (3)	0.0056 (3)
C4	0.0161 (3)	0.0160 (3)	0.0135 (3)	0.0033 (2)	0.0044 (2)	0.0052 (2)
C5	0.0180 (3)	0.0170 (3)	0.0150 (3)	0.0018 (3)	0.0053 (3)	0.0055 (3)
C6	0.0181 (3)	0.0174 (3)	0.0151 (3)	0.0021 (3)	0.0042 (3)	0.0045 (3)
C7	0.0154 (3)	0.0174 (3)	0.0144 (3)	0.0038 (2)	0.0042 (2)	0.0052 (3)
C8	0.0155 (3)	0.0177 (3)	0.0125 (3)	0.0041 (2)	0.0039 (2)	0.0049 (2)
C9	0.0213 (3)	0.0203 (3)	0.0183 (3)	0.0079 (3)	0.0100 (3)	0.0076 (3)
C10	0.0228 (4)	0.0201 (3)	0.0193 (4)	0.0075 (3)	0.0085 (3)	0.0077 (3)
C11	0.0210 (3)	0.0244 (4)	0.0129 (3)	0.0055 (3)	0.0062 (3)	0.0061 (3)
C12	0.0215 (3)	0.0205 (3)	0.0122 (3)	0.0068 (3)	0.0056 (3)	0.0041 (3)
C13	0.0254 (4)	0.0250 (4)	0.0209 (4)	0.0003 (3)	0.0053 (3)	0.0124 (3)
Cl1	0.01988 (9)	0.01675 (9)	0.01702 (9)	0.00538 (6)	0.00752 (7)	0.00529 (7)
O4	0.0248 (3)	0.0491 (5)	0.0350 (4)	−0.0025 (3)	0.0119 (3)	0.0193 (4)
O5	0.0544 (5)	0.0345 (4)	0.0236 (4)	0.0117 (4)	0.0172 (4)	−0.0019 (3)
O6	0.0580 (6)	0.0274 (4)	0.0320 (4)	0.0249 (4)	0.0095 (4)	0.0123 (3)
O7	0.0185 (3)	0.0273 (3)	0.0383 (4)	0.0036 (2)	0.0036 (3)	0.0102 (3)

### Geometric parameters (Å, °)

**Table:**

O1—N1	1.2280 (10)	C6—H6	0.9500
O2—N1	1.2260 (10)	C7—C8	1.5052 (11)
O3—C7	1.2191 (10)	C8—C12	1.3910 (11)
N1—C1	1.4655 (10)	C8—C9	1.3915 (11)
N2—C11	1.3466 (11)	C9—C10	1.3800 (12)
N2—C10	1.3487 (11)	C9—H9	0.9500
N2—C13	1.4803 (11)	C10—H10	0.9500
C1—C6	1.3855 (11)	C11—C12	1.3819 (12)
C1—C2	1.3858 (11)	C11—H11	0.9500
C2—C3	1.3885 (11)	C12—H12	0.9500
C2—H2	0.9500	C13—H13A	0.9800
C3—C4	1.3988 (11)	C13—H13B	0.9800
C3—H3	0.9500	C13—H13C	0.9800
C4—C5	1.3984 (11)	Cl1—O4	1.4337 (8)
C4—C7	1.4885 (11)	Cl1—O6	1.4340 (8)
C5—C6	1.3911 (11)	Cl1—O5	1.4383 (8)
C5—H5	0.9500	Cl1—O7	1.4401 (8)
			
O2—N1—O1	124.08 (8)	C12—C8—C9	119.35 (7)
O2—N1—C1	118.26 (7)	C12—C8—C7	118.31 (7)
O1—N1—C1	117.66 (7)	C9—C8—C7	122.32 (7)
C11—N2—C10	121.28 (7)	C10—C9—C8	119.22 (7)
C11—N2—C13	119.58 (7)	C10—C9—H9	120.4
C10—N2—C13	119.09 (8)	C8—C9—H9	120.4
C6—C1—C2	123.63 (7)	N2—C10—C9	120.44 (8)
C6—C1—N1	118.23 (7)	N2—C10—H10	119.8
C2—C1—N1	118.14 (7)	C9—C10—H10	119.8
C1—C2—C3	117.82 (7)	N2—C11—C12	120.44 (7)
C1—C2—H2	121.1	N2—C11—H11	119.8
C3—C2—H2	121.1	C12—C11—H11	119.8
C2—C3—C4	120.07 (7)	C11—C12—C8	119.18 (8)
C2—C3—H3	120.0	C11—C12—H12	120.4
C4—C3—H3	120.0	C8—C12—H12	120.4
C5—C4—C3	120.66 (7)	N2—C13—H13A	109.5
C5—C4—C7	121.64 (7)	N2—C13—H13B	109.5
C3—C4—C7	117.66 (7)	H13A—C13—H13B	109.5
C6—C5—C4	119.76 (7)	N2—C13—H13C	109.5
C6—C5—H5	120.1	H13A—C13—H13C	109.5
C4—C5—H5	120.1	H13B—C13—H13C	109.5
C1—C6—C5	118.03 (7)	O4—Cl1—O6	110.12 (6)
C1—C6—H6	121.0	O4—Cl1—O5	109.24 (6)
C5—C6—H6	121.0	O6—Cl1—O5	108.89 (6)
O3—C7—C4	121.94 (7)	O4—Cl1—O7	109.05 (5)
O3—C7—C8	118.68 (7)	O6—Cl1—O7	109.59 (5)
C4—C7—C8	119.37 (7)	O5—Cl1—O7	109.94 (5)
			
O2—N1—C1—C6	−36.02 (11)	C5—C4—C7—C8	−23.70 (11)
O1—N1—C1—C6	143.63 (8)	C3—C4—C7—C8	158.50 (8)
O2—N1—C1—C2	144.34 (8)	O3—C7—C8—C12	−39.66 (11)
O1—N1—C1—C2	−36.01 (11)	C4—C7—C8—C12	139.93 (8)
C6—C1—C2—C3	0.10 (13)	O3—C7—C8—C9	138.36 (9)
N1—C1—C2—C3	179.72 (7)	C4—C7—C8—C9	−42.06 (11)
C1—C2—C3—C4	−1.68 (13)	C12—C8—C9—C10	−2.33 (13)
C2—C3—C4—C5	1.70 (13)	C7—C8—C9—C10	179.68 (8)
C2—C3—C4—C7	179.52 (8)	C11—N2—C10—C9	2.39 (13)
C3—C4—C5—C6	−0.10 (13)	C13—N2—C10—C9	−175.05 (8)
C7—C4—C5—C6	−177.83 (7)	C8—C9—C10—N2	−0.59 (13)
C2—C1—C6—C5	1.46 (13)	C10—N2—C11—C12	−1.20 (13)
N1—C1—C6—C5	−178.16 (7)	C13—N2—C11—C12	176.23 (8)
C4—C5—C6—C1	−1.43 (12)	N2—C11—C12—C8	−1.75 (13)
C5—C4—C7—O3	155.88 (8)	C9—C8—C12—C11	3.47 (12)
C3—C4—C7—O3	−21.92 (12)	C7—C8—C12—C11	−178.45 (7)

### Hydrogen-bond geometry (Å, °)

**Table:**

*D*—H···*A*	*D*—H	H···*A*	*D*···*A*	*D*—H···*A*
C2—H2···O7^i^	0.95	2.60	3.478 (1)	153.
C3—H3···O7^ii^	0.95	2.42	3.277 (1)	150.
C5—H5···O4	0.95	2.51	3.394 (1)	154.
C9—H9···O7^iii^	0.95	2.42	3.132 (1)	132.
C10—H10···O3^iv^	0.95	2.52	3.392 (1)	153.
C11—H11···O1^v^	0.95	2.43	3.200 (1)	138.
C12—H12···O5^vi^	0.95	2.39	3.134 (1)	135.
C13—H13A···O3^iv^	0.98	2.63	3.373 (1)	133.
C13—H13B···O6^vii^	0.98	2.59	3.429 (1)	144.
C13—H13C···O2^viii^	0.98	2.63	3.446 (1)	141.
C13—H13C···O6	0.98	2.60	3.402 (1)	139.

Symmetry codes: (i) −*x*+1, −*y*, −*z*+1; (ii) *x*−1, *y*−1, *z*; (iii) *x*−1, *y*, *z*; (iv) *x*, *y*+1, *z*; (v) *x*, *y*, *z*−1; (vi) −*x*+1, −*y*, −*z*; (vii) −*x*+1, −*y*+1, −*z*; (viii) −*x*+1, −*y*+1, −*z*+1.
